# Expression pattern of CDK12 protein in gastric cancer and its positive correlation with CD8^+^ cell density and CCL12 expression

**DOI:** 10.7150/ijms.34541

**Published:** 2019-08-06

**Authors:** Jun Ji, Chenfei Zhou, Junwei Wu, Qu Cai, Min Shi, Huan Zhang, Yingyan Yu, Zhenggang Zhu, Jun Zhang

**Affiliations:** 1 Shanghai Institute of Digestive Surgery, Ruijin Hospital, Shanghai Jiao Tong University School of Medicine, No. 197 Ruijin er Road, Shanghai, 200025, China.; 2 Department of Oncology, Ruijin Hospital, Shanghai Jiao Tong University School of Medicine, No. 197 Ruijin er Road, Shanghai, 200025, China.; 3 Department of Radiology, Ruijin Hospital, Shanghai Jiao Tong University School of Medicine, No. 197 Ruijin er Road, Shanghai, 200025, China.

**Keywords:** Gastric cancer, CDK12, lymph node metastasis, CD8^+^ cells, CCL21

## Abstract

**Background:** The aims of this study were to investigate the expression pattern of CDK12 protein in gastric cancer, and to analyze the correlations of CDK12 expression between CD8^+^ cell density and CCL12 expression.

**Methods:** Eighty-six paired tumor and non-tumor samples were collected from patients who underwent radical surgery and had pathological confirmed gastric adenocarcinoma. Immunohistochemistry was used to assess CDK12 expression and CD8^+^ cell density. Expression of CDK12 and CCL21 mRNA was detected by quantitative reverse transcription-polymerase chain reaction.

**Results:** CDK12 expression in gastric tumor tissues was significantly higher than it in paired non-tumor tissues (P<0.001). High expression of CDK12 was identified in 43 cases (50%), and it was significantly correlated with Lauren's classification (diffuse type) and number of metastatic lymph nodes (≥15). High CDK12 protein level indicated a relative poorer overall survival than patients with CKD12 low expression, while it was not identified as an independent prognostic factor. Median number of CD8^+^ cells in tumor tissues was 51 (range: 0-292). Number of CD8^+^ cells was positively correlated with CDK12 expression score in tumor tissues (r=0.243, P=0.024). Positive correlation was also found between CDK12 and CCL21 mRNA expression (r=0.419, P=0.017).

**Conclusion:** High CDK12 expression was detected in gastric cancer which was correlated with malignant phenotypes and worse outcome. Positive correlations of CD8^+^ cell number and CCL21 mRNA expression with CDK12 level were identified.

## Introduction

Gastric cancer is one of the most prevalent malignant diseases in eastern Asia^1^. Five-year overall survival rate of gastric cancer patients in China is only about 30%^2^. Its high cancer-related mortality is mainly caused by tumor recurrence and distant metastasis. Chemotherapy is still the major strategy to treat gastric cancer patients with late stage^3^. For patients with epidermal growth factor receptor 2 (HER2) overexpression, the efficacy of anti-HER2 monoclonal antibody trastuzuamb in combination with chemotherapy has been demonstrated by randomized phase III clinical trial^4^.

In clinical practices, patients with HER2 overexpression who are poorly responded to trastuzuamb treatment can be found, which indicates the existence of primary resistant mechanisms. To explore unknown driver molecular events in HER2 positive gastric cancer, whole-genome sequencing analysis comparing HER2 positive and negative gastric cancer was performed and analyzed in our pervious investigation. The results showed that cyclin dependent kinase 12 (CDK12) gene amplification was mainly detected in HER2 positive patients, and it was also identified as one of the driver molecular events in gastric cancer^5^.

Co-amplification of *CDK12* with *HER2* has been reported in HER2 positive breast cancer^6^. Furthermore, genomic alterations and aberrant expression of CDK12 are found in multiple cancers, and it also participates in regulating tumor malignant phenotypes^7^. In our pervious study, we first reported amplification of *CDK12* in HER2 positive gastric cancer. However, expression pattern of CDK12 protein in gastric cancer has not been investigated.

In present study, we aimed to detect the expression of CDK12 protein in gastric cancer and to explore its correlation with patients' clinicopathological features. Furthermore, it was reported that *CDK12* loss in prostate cancer was associated with genomic instability and tandem duplication which leaded to increased T cell infiltration^8^. CD8^+^ T cells are the major effect cells in anti-tumor immunity. Therefore, correlation of CDK12 expression with CD8^+^ cell density in gastric cancer was investigated, as well as CC-chemokine ligand 21 (CCL21) expression, a chemokine which can induce T cell infiltration.

## Materials and methods

### Patients and samples

Samples of 86 gastric cancer patients underwent standard D2 resection in Department of Surgery, Shanghai Ruijin Hospital were collected. All samples were confirmed as gastric adenocarcinoma by pathological diagnosis. Information of clinicopathological characteristics and survival status of all patients were recorded. TNM staging classification was based on criteria of American Joint Committee on Cancer (AJCC, 8^th^ edition).

### Immunohistochemistry staining (IHC)

Tissue microarray was made by paraffin-embedded specimens. Immunohistochemistry staining was performed following procedure of Dako REAL Envision Detected System (Dako), Primary antibodies including CDK12 (Proteintech, 26816-1-AP, 1:200) and CD8 (Dako, M7103, 1:100) were used. HRP labeled secondary antibody was used and was visualized by diaminobenzidine.

CDK12 staining was localized in cell nuclear. The expression status of CDK12 was determined by sum score of intensity and percentage of positive cells under high-power field with typical staining. Score for percentage: absent (0); background (1); 1%-25% (2); 26%-50% (3); 51%-75% (4); >75% (5); for intensity: no staining (0); light (1); moderate (2); strong (3). Score of 7 to 8 were identified as high expression, and 0 to 6 as low expression^9^. CD8 stained cells were counted by Image-Pro Plus software. Three random fields (amplification 200×) on each core were selected and average number of CD8^+^ cells was calculated. Number of stained cells higher than 35 was identified as high density otherwise was identified as low density^10^.

### Quantitative reverse transcription-polymerase chain reaction (qRT-PCR)

Total tissue RNA was extracted by Trizol reagent method. Reverse transcription in 20μl-system was performed following protocol of Applied Biosystems. Primers for qRT-PCR were CDK12 (forward primer: CTA ACA GCA GAG AGC GTC ACC; reverse primer: AAA GGT TTG ATA ACT GTG CCC A) and CCL21 (forward primer: GTT GCC TCA AGT ACA GCC AAA; reverse primer: AGA ACA GGA TAG CTG GGA TGG). Real-time PCR was performed by ABI prism 7900HT sequence detection system (Applied Biosystems). Relative mRNA expression was calculated by comparative Ct method.

## Statistics

Expression of CDK12 in paired tissues was analyzed by paired-samples nonparametric test. Correlation of CDK12 expression with clinicopathological characteristics of gastric cancer patients were analyzed by Pearson's χ^2^ test. Spearman test was used to assess correlation analysis. Log-rank test in Kaplan-Meier method and Cox regression test were used to analyze prognostic factors. Independent-sample *t* test was used to analyze quantitive data of mRNA expression. *P*-value <0.05 was considered as statistically significant. All tests were performed by SPSS 22.0 software (SPSS Inc.).

## Results

### Clinicopathological characteristics of 86 gastric cancer patients

Clinicopathological characteristics of all enrolled gastric cancer patients were listed in Table 1. There were 59 male patients (68.6%) and 27 female patients (31.4%), with median age of 63 years old. For Lauren's classification, patients with intestinal type and diffuse type were both 43 cases. Most patients were pathological stage III (54 cases, 62.8%).

### CDK12 protein expression in gastric cancer tissues

**Figure 1:**
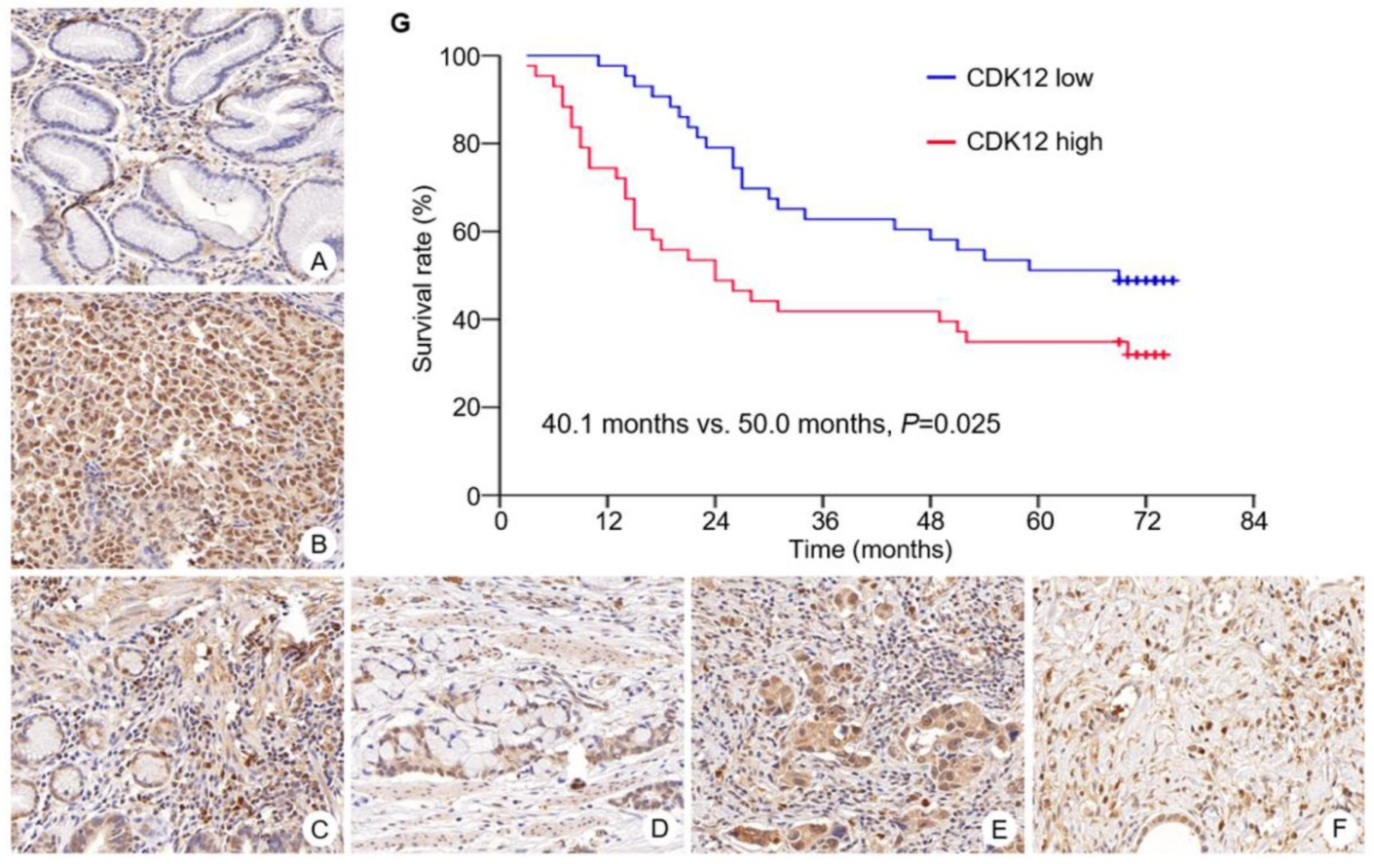
** Expression of CDK12 in gastric cancer and survival curve of patients with different CDK12 expression.** CDK12 was located in cell nuclear. Expression of CDK12 in non-tumor tissue (A) was lower than its in paired tumor tissue (B). Representative images of CDK12 staining with different score were showed. The intensity scores of image C to F were all 3. The percentage scores were (C) 2; (D) 3; (E) 4; and (F) 5. All images at 400× magnification. G: Patients with CDK12 high expression had a shorter median overall survival than those with CDK12 low expression (40.1 months vs. 50.0 months, *P*=0.025).

Immunohistochemistry staining of CDK12 was performed in all gastric cancer tissues and paired non-tumor tissues (Figure 1). Median scores of CDK12 in tumor tissues and non-tumor tissues were 6 (range: 3-8) and 5 (range: 0-8), respectively. Staining scores of CDK12 in tumor tissues were significantly higher than those in paired non-tumor tissues (*P*<0.001).

In tumor tissues, high expression of CDK12 was identified in 43 cases (50%). Its high expression was correlated with Lauren's classification (diffuse type, *P*=0.005) and lymph node metastasis (number of metastatic lymph nodes ≥15, *P*=0.041) (Table 2). Correlation of CDK12 expression with patients' gender, age, tumor site, T stage, N stage and TNM stage was not found.

Overall survival of patients with CDK12 high expression was significantly shorter than those with CDK12 low expression (median survival: 40.1 months vs. 50.0 months, *P*=0.025) (Figure 1G). By multivariate analysis, number of metastatic lymph node ≥15 was identified as an independent prognostic factor (HR 2.90, 95%CI 1.152 to 7.296, *P*=0.024).

### Correlation between CDK12 expression and CD8^+^ cell number in gastric cancer

**Figure 2:**
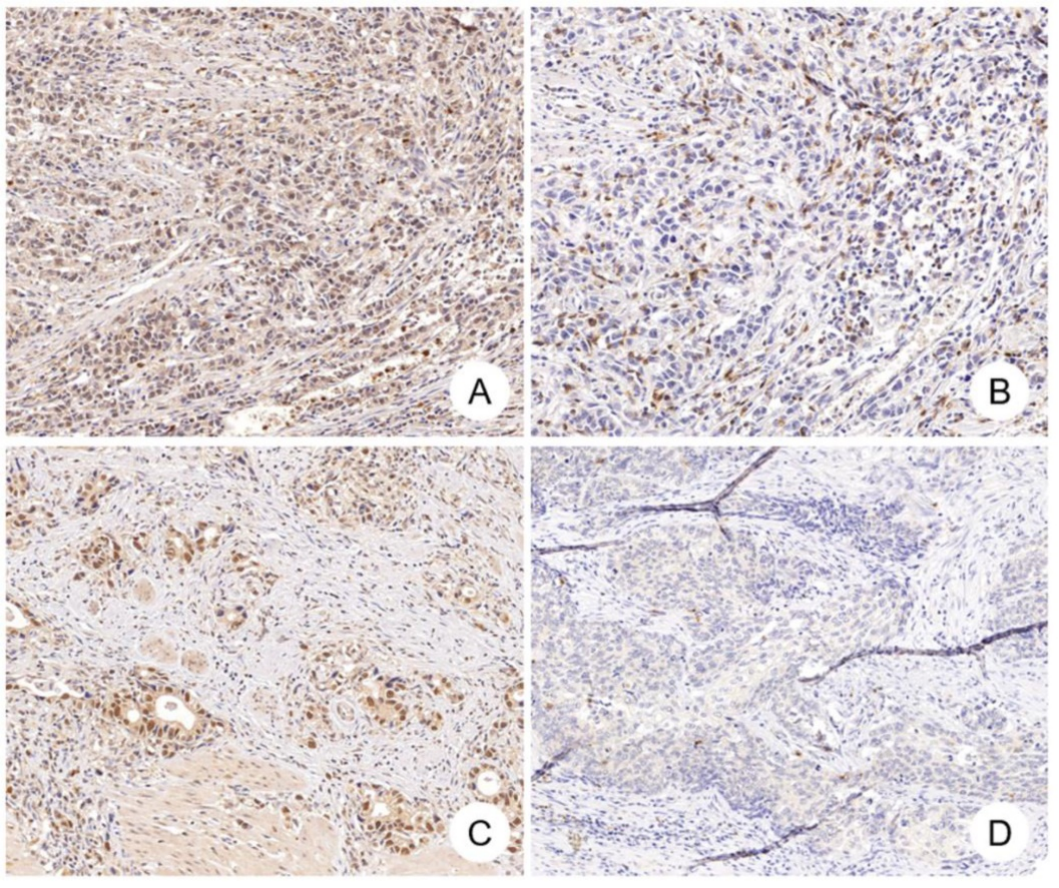
** CDK12 expression and CD8^+^ cells in gastric cancer.** Positive correlation between CDK12 expression and CD8^+^ cells number was identified in gastric cancer. CDK12 high expression **(A)** and CD8^+^ cells number over 35 **(B)** were detected in one gastric tumor sample, while CDK12 low expression **(C)** and CD8^+^ cells number less than 35 **(D)** in another tumor sample.

**Figure 3:**
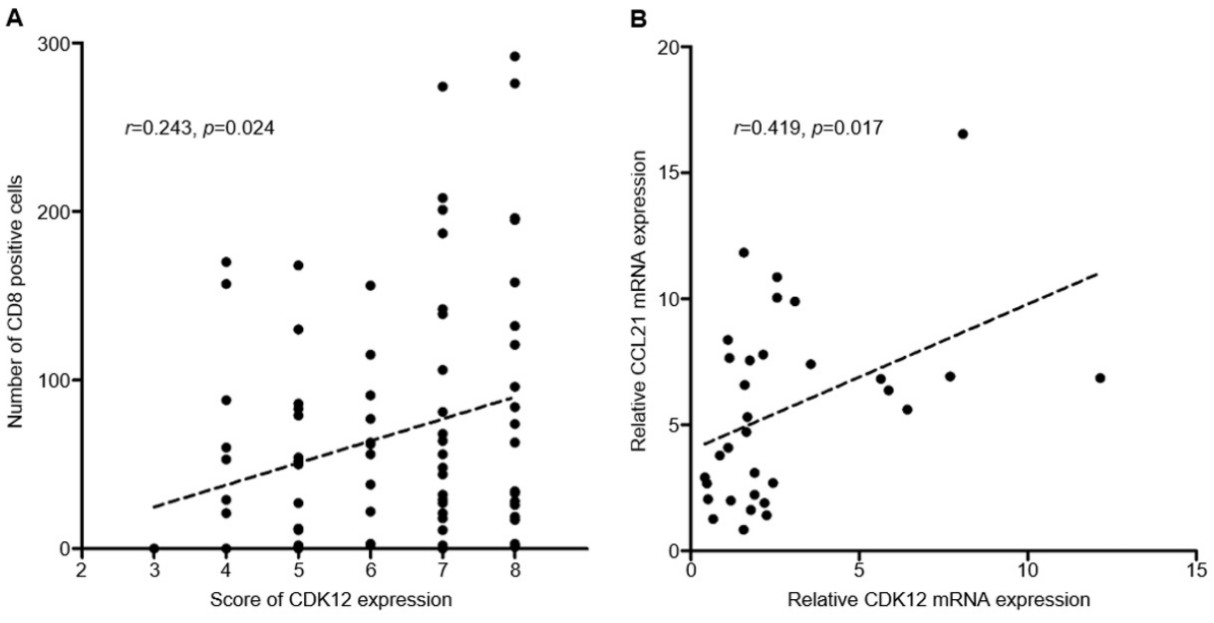
** Correlation of CDK12 with number of CD8^+^ cells and CCL21. A:** Correlation analysis between CDK12 staining score and number of CD8^+^ cells; **B:** Correlation analysis between CDK12 and CCL21 mRNA expression.

Median number of CD8^+^ cells in tumor tissues was 51 (range: 0-292). Positive correlation was found between CDK12 expression score and number of CD8^+^ cells in tumor tissues (*r*=0.243, *P*=0.024) (Figure 2, Figure 3A). High CD8^+^ cell density was identified in 47 cases (54.7%). Correlation of CD8^+^ cell density with patients' clinicopathological characteristics and overall survival was not found.

### Correlation between CDK12 and CCL21 mRNA expression

CC-chemokine ligand 21 (CCL21) is one of the chemokines which can induce T cell infiltration, and its expression was reported changed in *CDK12* mutant tumors. To analyze the correlation between CCL21 and CDK12 in gastric cancer, expression of CDK12 and CCL21 mRNA were detected in 16 gastric cancer tissues and their paired non-tumor tissues. Similar with IHC staining, mRNA expression of CDK12 in tumor tissues was significantly higher than that in non-tumor tissues (4.04±3.25 vs. 1.56±0.84, *P*=0.009). Expression of CCL21 was not different between tumor and non-tumor tissues (6.56±4.27 vs. 4.65±2.75, *P*=0.140). Positive correlation was found between CDK12 and CCL21 mRNA expression (*r*=0.419, *P*=0.017) (Figure 3B).

## Discussion

In present study, we found that CDK12 was overexpressed in gastric cancer tissues, and its expression was positively correlated with Lauren's classification and high number of metastatic lymph nodes. High expression of CDK12 indicated a relatively poor outcome, although was not identified as an independent prognostic factor. Positive correlations were found between CDK12 expression and number of CD8^+^ cells in tumor tissues, as well as between CDK12 and CCL21 mRNA expression.

*CDK12* gene is located on chromosome 17q12 which is about 200kb proximal to the *ERBB2* gene. Therefore, *ERBB2* gene amplification often co-occurs with *CDK12* gene amplification. In breast cancer, more than 70% of *ERBB2* amplicon contained *CDK12* gene^11^. In our previous study, *CDK12* amplification was also detected in 60% of HER2 positive gastric cancer^5^. Genomic alterations of CDK12 were also detected in multiple malignant diseases including oesophageal, stomach, breast, colorectal and ovarian cancer with ranging from 5% to 15% of detected cases 7. However, protein expression of CDK12 was only analyzed in breast cancer. The results showed that CDK12 high expression rate was 21%, and its absent rate was higher in triple-negative breast cancer tissues^9^. In present study, absent of CDK12 expression was not detected in gastric cancer tissues. High expression rate of CDK12 was 50%, which was much higher than previously reported CDK12 amplification rate in gastric cancer. High expression of CDK12 mRNA in tumor tissues was also detected in our study. These results indicate that in addition to genomic alterations, transcriptional or translational process also play important role in regulating CDK12 expression in gastric cancer.

CDK12 belongs to the transcriptional subfamily of cyclin-dependent kinases (CDKs), which binds Cyclin K (CycK) to form complex that can phosphorylate Ser2 in C-terminal domain of RNA polymerase II, thereby participating in regulating transcription of specific subset of human genes. DNA damage response genes including *BRCA1* (breast and ovarian cancer type 1 susceptibility protein 1), *ATR* (ataxia telangiectasia and Rad3-related), *FANCI* (FA complementation group I) and *FANCD2* (FA complementation group D2) were its known target genes^12^. *CDK12* depleted embryos dead shortly after implantation due to genomic instability and pluripotency loss of embryonic stem cells^13^. Therefore, the previous investigations of biological function of CDK12 in cancer cells were mainly focused on its ability to maintain DNA repair pathways. Loss of CDK12 function increased sensitivity of ovarian cancer cells to cisplatin and poly (ADP-ribose) polymerase (PARP) inhibitors^14^. Synthetic lethal effect could be detected when CDK12 inhibitors were administrated combining with MYC, EWS/FLI and PARP inhibitors^[15-17]^.

Currently, the biological functions of CDK12 in gastric cancer have not been fully investigated. Positive correlation of CDK12 high expression with Lauren's classification, especially with high number of metastatic lymph nodes suggested that the functions of CDK12 might not be limited to maintain genomic stability of cancer cells. In breast cancer, alternative last exon (ALS) splicing of genes with long transcripts and many exons can be regulated by CDK12. Depletion of CDK12 in breast cancer cells resulted in accumulation of DNAJB6 long isoform, thereby decreasing the migration and invasion abilities of MDA-MB-231 cells^18^. In osteosarcoma cells, CDK12 could also play a role in transcriptional regulation of the MAP3K14 and NFKB2 mRNA expression, participating in activation of noncanonical NF-κB pathway^19^. Therefore, RNA processing or transcriptional regulation of downstream effect genes can be one of the mechanisms of CDK12 regulating biological behavior of cancer cells.

Recently, in prostate cancer, *CDK12* biallelic inactivating mutation was significantly correlated with T cells infiltration and identified a novel subtype of metastatic castration-resistant prostate cancer with high sensitivity to immune checkpoint inhibition^8^. The efficacy of immunotherapy in gastric cancer has been demonstrated, and patients with EBV (Epstein-Barr virus) type according to comprehensive molecular characterization might be more sensitive to immune checkpoint inhibitors^3^. Whether CDK12 protein expression is correlated with infiltration of immune cells in gastric cancer has not been investigated. In present study, positive correlation between CDK12 expression and CD8^+^ cell density was detected. In tumor tissues with CDK12 high expression, a relatively high CD8^+^ cell density was found. This result appeared to contradict to current evidence. Different detection level of CDK12 in our study comparing with pervious investigation suggested that genomic alterations of *CDK12* might not fully represent its protein function. RNA processing or transcriptional effect of CDK12 other than genomic instability maintenance might be the dominant mechanisms to induce T cell infiltration in CDK12 high expression gastric cancer. Therefore, the potential downstream effect genes regulated by CDK12 should be investigated in gastric cancer.

Different expression of inflammatory gene signature was found in *CDK12* mutant tumors, including CCL21 which can induce T cells and dendritic cells infiltration^[8, 20]^. Therefore, CCL21 was investigated here as a potential downstream effector of CDK12. By qRT-PCR, positive correlation between mRNA expression of CDK12 and CCL21 was found in present study. CCL21 can both regulate biological functions of tumor cells and immune cells. Activation of CCL21/CCR7 axis could induce tumor progression and metastasis in melanoma, breast cancer and colorectal cancer^21^. In gastric cancer, high expression of CCL21 in tumor tissues was correlated with tumor invasion and lymph node metastasis^22^. Induction of CCL21 was found in tertiary lymphoid structures which participated in development of inflammation-associated cancer in spontaneous gastric tumorigenesis model^23^. For immune cells, CCL21 can induce several processes of immune response activation including co-localization and recruitment of T cells and dendritic cells; improving cell migration; co-stimulation of naïve T cell expansion and Th1 polarization^21^. However, inhibitory program triggered in T cells after treatment of high concentration of CCL21 was also observed^24^. Regulatory CD8^+^ T cells which suppressed tumor associated antigen-specific T cell priming was activated by CCR7 pathway^25^. From the results of present study, CCL21 could be one of the potential downstream effect genes of CDK12 in gastric cancer. High level of CCL21 in CDK12 high expression tumors may promote lymph node metastasis and increase inflation of CD8^+^ T cells. On the other hand, immune activation of T cells was inhibited by consistent high concentration of CCL21 which resulted in the poor outcome of patients with high CDK12 expression. Therefore, further investigations should be performed to validate the hypothesis of CDK12/CCL21 pathway in regulation malignant function of tumor cells and immune microenvironment in gastric cancer, as well as the mechanisms of CDK12 regulating CCL21 expression.

## Conclusion

To summary, high expression of CDK12 protein was detected in gastric cancer and was correlated with malignant phenotypes and worse outcome. Positive correlations of CD8^+^ cell number and CCL21 mRNA expression with CDK12 were identified, which indicated the potential mechanisms of CDK12 regulating biological behavior of gastric cancer.

## Figures and Tables

**Table 1:** Pathological characteristics of gastric cancer patients

Clinicopathological characteristics		N (n=86)	Percent (%)
Gender	Male	59	68.6
	Female	27	31.4
Age	Median (range)	63 (32-89)	
Site	Cardia	8	9.3
	Corpus	29	33.7
	Antrum	49	57.0
Lauren's	Intestinal	43	50.0
	Diffuse	43	50.0
T stage	T1	3	3.5
	T2	12	14.0
	T3	46	53.5
	T4	25	29.1
N stage	N0	19	22.1
	N1	17	19.8
	N2	24	27.9
	N3a	19	22.1
	N3b	7	8,1
TNM stage	I	10	11.6
	II	22	25.6
	IIIA	27	31.4
	IIIB	19	22.1
	IIIC	8	9.3

**Table 2:** Correlation between CDK12 expression and patients' clinicopathological characteristics

Clinicopathological characteristics		CDK12		P value
		Low (%)	High (%)	
Gender				
	Male	30 (50.8)	29 (49.2)	0.816
	Female	13 (48.1)	14 (51.9)	
Age				
	<60	20 (60.6)	13 (39.4)	0.121
	≥60	23 (43.4)	30 (56.6)	
Site				
	Cardia	4 (50.0)	4 (50.0)	0.258
	Corpus	11 (37.9)	18 (62.1)	
	antrum	28 (57.1)	21 (42.9)	
Lauren's				
	Intestinal	28 (65.1)	15 (34.9)	0.005
	Diffuse	15 (34.9)	28 (65.1)	
T stage				
	T1	3 (100.0)	0 (0)	0.073
	T2-3	30 (51.7)	28 (48.2)	
	T4	10 (40.0)	15 (60.0)	
N stage				
	N0	12 (63.2)	7 (36.8)	0.537
	N1	9 (52.9)	8 (47.1)	
	N2	11 (45.8)	13 (54.2)	
	N3	11 (42.3)	15 (57.7)	
Lymph node metastasis				
	0	12 (63.2)	7 (36.8)	0.041
	1-14	30 (50.8)	29 (49.2)	
	≥15	1 (12.5)	7 (87.5)	
TNM stage				
	I-II	20 (62.5%)	12 (37.5)	0.073
	III	23 (42.6%)	31 (57.4)	

## Abbreviations

HER2: epidermal growth factor receptor 2
CDK12: cyclin dependent kinase 12
IHC: Immunohistochemistry staining
qRT-PCR: Quantitative reverse transcription-polymerase chain reaction
CCL-21: CC-chemokine ligand 21
CDKs: cyclin-dependent kinases
CycK: Cyclin K
BRCA1: breast and ovarian cancer type 1 susceptibility protein 1
FANCI: FA complementation group I
ATR: ataxia telangiectasia and Rad3-related
FANCD2: FA complementation group D2
EBV: Epstein-Barr virus
